# Synthesis, Vasodilating, Antithrombotic and Cardioprotective Activity of Pyridyl Substituted 5-Silyl(Germyl)Isoxazolines-2

**DOI:** 10.1155/MBD.1998.251

**Published:** 1998

**Authors:** E. Lukevics, P. Arsenyan, M. Veveris

**Affiliations:** Latvian Institute of Organic Synthesis Aizkraukles 21 Riga LV-1006 Latvia

## Abstract

The reactions of [2+3] cycloaddition of pyridylnitrile oxides to vinyl- and allylgermanes proceed regioselectively and afford 5-Ge-substituted isoxazolines-2. We have synthesized 9 new pyridyl substituted 5-Si(Ge)-isoxazolines-2 and investigated their biological activity. The vasodilating, anticoagulant and cardioprotective activities of 5-Si(Ge) substituted isoxazolines-2 have been studied in *vitro* and in *vivo*. Substitution of the silicon atom for the germanium one leads to the significant increase in vasodilating, antithrombotic and cardioprotective activity. The insertion of the methylene group between Ge and the isoxazoline ring reduces the vasodilating activity. The most active isoxazoline - 3-(5‵-triethylgermyl-3‵-isoxazolinyl)pyridine hydrochloride protects the heart from rhythm disturbances and lethality during ischaemia-reperfusion.


### SYNTHESIS, VASODILATING, ANTITHROMBOTIC AND CARDIOPROTECTIVE ACTIVITY OF PYRIDYL SUBSTITUTED 5-SILYL(GERMYL)ISOXAZOLINES-2

E. Lukevics*, P. Arsenyan, and M. Veveris

Latvian Institute of Organic Synthesis, Aizkraukles 21, Riga, LV-1006, Latvia

### ABSTRACT

The reactions of [2+3] cycloaddition of pyridylnitrile oxides to vinyl- and allylgermanes proceed regioselectively and afford 5-Ge-substituted isoxazolines-2. We have synthesized 9 new pyridyl substituted 5-Si(Ge)-isoxazolines-2 and investigated their biological activity. The vasodilating, anticoagulant and cardioprotective activities of 5-Si(Ge) substituted isoxazolines-2 have been studied in *vitro* and in *vivo*. Substitution of the silicon atom for the germanium one leads to the significant increase in vasodilating, antithrombotic and cardioprotective activity. The insertion of the methylene group between Ge and the isoxazoline ring reduces the vasodilating activity. The most active isoxazoline - 3-(5`-triethylgermyl-3`-isoxazolinyl)pyridine hydrochloride protects the heart from rhythm disturbances and lethality during ischaemia-reperfusion.

### INTRODUCTION

In 1994 the first organogermanium pharmaceutical propagermanium was launched in Japan under the trade name Serocion® (Sanwa Kenkyusho Co.,Ltd.). Its biological activity spectrum includes the protection against viruses, immunostimulation and hepatoprotection. Propagermanium has been introduced into clinics for the treatment of chronic hepatitis. This compound belonging to germsesquioxanes has been shown to possess low toxity.

This achievement has stimulated the further investigations of the biological activity not only of germsesquioxanes but also of other classes of low toxic organogermanium compounds.

Hetarylgermatranes show relatively high neurotropic activity [1, 2]. Triphenylgermyl substituted 1,2,4-triazolinones were effective against gastric carcinoma MGC-803 under the experimental conditions. However, no inhibitory effects were found against carcinoma BGC-823 [3].

[2+3] Cycloaddition reactions of nitrile oxides to vinylsilanes proceed regiospecifically to give 5-Si substituted isoxazolines-2 [4-8], however, the addition to vinyltriethoxysilane and vinylsilatrane gives a mixture of 4- and 5-Si regioisomers [9]. Silyl substituted isoxazolines show wide spectrum of the biological activity [10].

The aim of the present work is the synthesis and investigation of vasodilating and cardioprotective activity of pyridyl substituted silyl- and germylisoxazolines.

### MATERIALS AND METHODS

### *Chemistry*

$^1$H NMR spectra were recorded on a Bruker WM-360/DS instrument at 360.1 MHz. GLC analysis was perfomed on a Varian 3700 instrument equipped with flame-ionizing detector using a capillar column 5 m × 0.53 mm, df=2.65 μ, HP-1. Carrier gas - nitrogen.

The melting points were determined on a ``Digital melting point analyzer`` (Fisher), the results are given without correction.

*General method of preparation.*

An equimolar amount of triethylamine in benzene (20 ml) was gradually added to a solution of vinyl(allyl)triethylsilane(germane) (0.02 mol) and pyridylhydroxamic chloride (0.02 mol) in benzene (30 ml) at 70° C. After addition of amine the mixture was stirred for 4 h at the same temperature. When the temperature of the reaction mixture fell to ambient, triethylamine hydrochloride was filtered off. The solvent was evaporated *in vacuo* and the residue was extracted with hexane. Hydrohalogenation was carried out by HHal in ether.

**Table 1**. Silyl(germyl)isoxazolines-2.

| | | |
|---|---|---|
| (1) | 2-(5`-triethylsilyl-3`-isoxazolinyl)pyridine hydrobromide | M.p.=198°C (from ethyl acetate). |
| (2) | 3-(5`-triethylsilyl-3`-isoxazolinyl)pyridine hydrobromide | M.p.=168°C (from ethyl acetate). |
| (3) | 4-(5`-triethylsilyl-3`-isoxazolinyl)pyridine hydrobromide | M.p.=181°C (from acetone/ ethanol (10:1)). |
| (4) | 2-(5`-triethylgermyl-3`-isoxazolinyl)pyridine hydrochloride | M.p.=115°C (from ethyl acetate/ methanol (2:1)). |
| (5) | 3-(5`-triethylgermyl-3`-isoxazolinyl)pyridine hydrochloride | M.p.=114°C (from acetone/ benzene (3:1)). |
| (6) | 4-(5`-triethylgermyl-3`-isoxazolinyl)pyridine hydrochloride | M.p.=148°C (from acetone). |
| (7) | 2-(5`-triethylgermylmethyl-3`-isoxazolinyl)pyridine hydrochloride | M.p.=102°C (from ethyl acetate/ methanol (4:1)). |
| (8) | 3-(5`-triethylgermylmethyl-3`-isoxazolinyl)pyridine hydrobromide | M.p.=148°C (from ethyl acetate/ 2-propanol (1:1)). |
| (9) | 4-(5`-triethylgermylmethyl-3`-isoxazolinyl)pyridine hydrobromide | M.p.=183°C (from ethyl acetate). |

### *Pharmacology*

*Vasodilating activity.* The modified classical method for the experiments on the isolated perfused rabbit ear blood vessels was used [11, 12]. Rabbits of both sexes (2.6-3.3 kg) were killed by *i.v.* injection of pentobarbital sodium (80 mg/kg). The central ear artery was dissected free at the base of the ear and cannulated with polyethylene tubing and perfused at a constant flow (2ml/min) from 4-channel peristaltic pump Geming (Italy).

The content of the perfusion fluid (mmol) was as follows: NaCl 136.9; KCl 2.68; $CaCl_2$ 1.8; $MgCl_2$ 1.05; $NaHCO_3$ 11.9; $NaH_2PO_4$ 0.42; glucose 5.6 (pH 7.35 at 22°C). Intraluminal inflow perfusion pressure was measured with a Statham P23J transducer and recorded on a physiograph DMP-4B (Narco Bio-Systems, USA). As flow remained constant, the changes in perfusion pressure reflected changes in blood resistance, i.e. the degree of vasoconstriction or relaxation. Vasoconstriction was caused by intraluminal infusion of noradrenaline (10 $\mu$mol). The investigated compounds were dissolved in perfusion fluid. The relaxant responses to the investigated compounds used in the different concentrations (10 and 50 $\mu$mol) were tested. Responses are expressed as per cent relaxation (% changes in the perfusion pressure) without and with the investigated compounds.

*Thrombos formation time.* Blood coagulation time was detected using Lee&White method [13]. White male mice of ICR-JCL strain (20-22 g) were used in the experiments (5 mice in every group). The studied compounds were administered i.p. 15 min prior the experiments. Blood was sampled from jugular vein from the previously anaesthetized mice (urethane 900 mg/kg, i.p.). The inhibition degree, the blood coagulation time for each compound in a dose of 30 mg/kg were expressed in min and in % ratio in comparison with the control (the blood coagulation time in the case of the solvent -0.6% solution of Tween 80).

*Blood bleeding time.* Blood bleeding time was measured following Duke [13]. White male mice of ICR-JCL strain (20-22 g) were used in the experiments (5 mice in every group). The studied compound or solvent (0.6% solution of Tween 80) was administered i.p. 15 min prior the test. The blood bleeding was caused from tail of the anasthetized mice (urethane 900 mg/kg, i.p.).

Table 2. Spectral and analytical data of compounds **1-9**.

| N° | Mol.Formula | ield (%) | 1H-NMR spectra(360.1MHz,$CDCl_3$/TMS, 303 K), δ(ppm); | Calc. (%)(found) N C H |
|---|---|---|---|---|
| *1* | $C_{14}H_{23}BrN_2OSi$ | 52 | 0.76 (6H, dq, J=1.8 Hz, J=5.6 Hz), 1.02(9H, t, J=5.6 Hz), 3.57 (1H, dd, J=10.2, J=21.9 Hz), 4.29 (1H, dd, J=10.2, J=21.9 Hz), 4.52(1H, dd, J=10.2 Hz, J=21.9 Hz), 7.98(1H, dt, J=2.1 Hz, J=5.6 Hz), 8.31-8.56 (2H, m), 9.00 (1H, dd, J=1.0 Hz, J=5.6 Hz). | 8.16(8.18) 48.96(49.05) 6.75(6.76) |
| *2* | $C_{14}H_{23}BrN_2OSi$ | 44 | 0.73 (6H, dq, J=1.8 Hz, J=5.8 Hz), 1.06(9H, t, J=5.8 Hz), 3.23(1H, dd, J=11.6 Hz, J= 15.6 Hz), 3.69 (1H, dd, J=11.6 Hz, J=15.6 Hz), 4.42(1H, dd, J=11.6 Hz, J=15.6 Hz), 8.04-8.17 (1H, m), 8.89-9.2 (3H, m). | 8.16(8.22) 48.96(49.07) 6.75(6.83) |
| *3* | $C_{14}H_{23}BrN_2OSi$ | 44 | 0.78 (6H, dq, J=2.1 Hz, J=6.2 Hz), 1.05 (9H, t, J=6.2 Hz), 3.42 (1H, dd, J=11.8 Hz,J=16.2 Hz), 3.83 (1H, dd, J=11.8 Hz, J=16.2Hz), 4.53 (1H, dd, J=11.8 Hz, J=16.2 Hz), 8.41 (2H, d, J=6.6 Hz), 9.05 (2H, d, J=6.6 Hz). | 8.16(8.20) 48.96(48.85) 6.75(6.81) |
| *4* | $C_{14}H_{23}ClGeN_2O$ | 70 | 0.96 (6H, q, J=6.7 Hz); 1.11 (9H, t, J=8.1 Hz); 3.54(1H, dd, J=12.0 Hz, J=17.2 Hz); 4.34 (1H, dd, J=12.0 Hz, J=15.7 Hz); 4.65 (1H, dd, J=15.7 Hz, J=17.2 Hz); 7.87 (1H, t, J=6.3 Hz); 8.34 (1H, td, J=0.7 Hz, J=1.3 Hz, J=8.3 Hz); 8.50 (1H, d, J=8.1Hz); 8.88 (1H, d, J=5.7 Hz). | 8.15(8.13) 48.98(48.92) 6.75(6.78). |
| *5* | $C_{14}H_{23}ClGeN_2O$ | 47 | 0.98 (6H, m); 1.11 (9H, m); 3.21 (1H, dd, J=11.4 Hz, J=15.5 Hz); 3.65 (1H, dd, J=11.4 Hz, J=15.5Hz), 4.45(1H, dd, J=11.4Hz, J=15.4 Hz); 8.01 (1H, dd, J=7.4 Hz, J=11.4 Hz); 8.81 (1H, m); 8.91 (1H, d, J=5 Hz). | 8.15(8.15) 48.98(48.99) 6.75(6.71). |
| *6* | $C_{14}H_{23}ClGeN_2O$ | 45 | 0.78-1.31 (15H, m); 3.20 (1H, dd, J=11.1 Hz, J=14.5 Hz); 3.64 (1H, dd, J=11.1Hz, J=14.5 Hz); 4.64 (1H, dd, J=11.1 Hz, J=14.5 Hz); 8.16 (2H, d, J=5.7 Hz); 8.9 (2H, d, J=5.7 Hz). | 8.15(8.08) 48.98(48.81) 6.75(6.72). |
| *7* | $C_{15}H_{25}ClGeN_2O$ | 43 | 0.88(6H,qd,J=1.5 Hz,J=7.2 Hz);1.07(9H, t,J=7.4 Hz); 1,28 (1H, dd, J=7.8 Hz, J=13.5 Hz); 1.41(1H, dd, J=7.8 Hz, J=13.5 Hz); 3.42 (1H, dd, J=9.4 Hz,J=17.7 Hz); 4.07 (1H, dd, J=10.5 Hz, J=17.7 Hz); 5.15 (1H,m); 7.83 (1H, t, J=6.5Hz); 8.31 (1H, m); 8.47 (1H, d, J=8.3 Hz); 8.84 (1H, d, J=5.0 Hz). | 7.84(7.82) 50.41(50.41) 7.05(7.06). |
| *8* | $C_{15}H_{25}BrGeN_2O$ | 45 | 0.76-1.22(15H,m);1.36(2H,t,J=10 Hz);2.97(1H, dd,J=10 Hz, J=17 Hz); 3.56 (1H, dd, J=10 Hz, J=17 Hz); 4.96-5.15 (1H, m); 8.0-8.15 (1H, m); 8.91-9.10 (3H, m). | 6.97(6.98) 44.83(44.85) 6.27(6.23). |
| *9* | $C_{15}H_{25}BrGeN_2O$ | 44 | 0.74-1.22 (15H, m); 1.35 (2H, t, J=8 Hz); 2.94 (1H, dd,J=9 Hz, J=16 Hz);3.51(1H, dd, J=9 Hz, J=16Hz); 5.13 (1H, m); 8.17 (2H, d, J=6 Hz); 8.98 (2H, d, J=6 Hz). | 6.97(6.98) 44.83(44.85) 6.27(6.27). |

*Heart failure produced by the coronary artery occlusion and reperfusion.* In acute trials on the anaesthetized (pentobarbital sodium, 50 mg/kg, i.p.) male Wistar rats (310-330 g) with artificial respiration (respirator V5kG Narco Bio-System, USA) the chest was open. A sling (6.0 silk Ethikon) was placed around the left coronary artery close to its origin and after recovery, the coronary artery was occluded for 10 min and reperfused (30 min). Incidence and duration of arrhythmia (ventricular tachycardia, fibrillation), ST-segment changes in ECG and mortality have been evaluated. All data were registered on a physiograph DMP-4B (Narco Bio-System, USA). The compounds were dissolved in 0.9% solution of NaCl and administered i.v. 5 min before the occlussion of coronary artery. The cardioprotective activity of the investigated compounds was compared with that of Allopurinol (xanthine oxidase inhibitor) which improved cardiac function and lowered hospital mortality rate in patients undergoing cardiac surgery [14].

*Acute toxicity.* The acute toxicity was evaluated in male ICR-JCL mice (19-23 g). The compounds were dissolved-suspended in 0.6% solution of Tween 80 and injected i.p.. To reduce the number of the used animals the maximal dose (400 mg/kg, i.p.) was used.

*Statistical analysis.* The results were presented as mean ± SEM for each group. The statistical analysis was perfomed using the Student's test for the unpaired data and Chi-square test. The results were considered as reliable at $p<0.05$.

### RESULTS AND DISCUSSION

### Chemistry

We have found that vinyltriethylgermane and allyltriethylgermane readily reacted with nitrile oxides giving the corresponding 5-Ge-isoxazolines-2 in relatively good yields (42-73%):

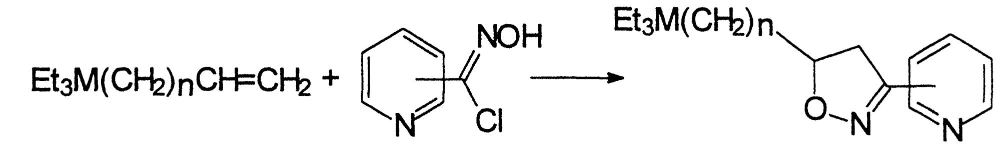

M=Si, Ge; n=0, 1.

Hydroxamic chlorides were obtained by the method described in Refs 15 and 16, and were transformed into cyanopyridine oxide [$Py\text{-}C{\equiv}N^{+}\text{-}O^{-}$] in the presence of triethylamine. The product formed interacted with vinyl(allyl)triethylgermane in the [2+3] cycloaddition reaction, affording the derivatives of 3-pyridyl-5-germyl substituted isoxazolines.

$^1H$ NMR data evidence that 5-triethylsilyl(germyl)-substituted isoxazoline is formed. The signals of two protons ($H_a$ and $H_b$) in 4 position of isoxazoline ring appear in the spectrum of compound ***1***: $H_a$ at 3.57(dd, $^2J$ =10.2 Hz -interaction with $H_b$, $^3J$=21.9 Hz - interaction with $H_c$) and $H_b$ at 4.29(dd, $^2J$ =10.2 Hz -interaction with $H_a$, $^3J$=21.9 Hz - interaction with $H_c$). $H_c$ in 5 position resonates in lower field as $\alpha$-substitutent is oxygen atom: 4.52(dd, $^3J$ =10.2 Hz, $^3J$=21.9 Hz).

$^1H$ NMR data for compound ***4*** are similar $H_a$ : 3.54(dd, $^2J$=12.0 Hz - interaction with $H_b$, $^3J$=17.2 Hz - interaction with $H_c$); $H_b$ : 4.34 (dd, $^2J$=12 Hz - interaction with $H_a$, $^3J$=15.7 Hz - interaction with $H_c$); $H_c$ : 4.65(dd, $^3J$=15.7 Hz - interaction with $H_b$, $^3J$=17.2 Hz - interaction with $H_a$).

$^1H$ NMR data for compound ***7*** differ to some extent due to the methylene group ($H_d$, $H_e$). $H_d$: 1.28 (dd, $^2J$=7.8 Hz - interaction with $H_e$, $^3J$=13.5 Hz - interaction with $H_c$), $H_e$ : 1.41 (dd, $^2J$=7.8 Hz - interaction with $H_d$, $^3J$=13.5 Hz - interaction with $H_c$). Protons $H_a$ and $H_b$ resonate like in compounds ***1*** and ***4***. $H_a$: 3.42 (dd, J=9.4 Hz, J=17.7 Hz), $H_b$ : 4.07 (dd, J=10.5 Hz, J=17.7 Hz). $H_c$ proton is shifted towards lower field (5.15, m).

Polarization of the vinyl group in triethylvinylgermane and triethylallylgermane is opposite and, consequently, one can expect the different regioselectivity of the nitrile oxides addition, while it has been proved experimentally that the addition occurs analogously [15-18].

**Table 4**. Vasodilating activity of the investigated compounds in noradrenaline-preconstricted rabbit's ear artery.

| Compound | Concentration (μ mol) | Relaxation (%) |
|---|---|---|
| 1 | 10 | ± 8 |
| | 50 | ± 15 |
| 2 | 10 | 23 |
| | 50 | 34* |
| 3 | 10 | 18 |
| | 50 | 32* |
| 4 | 10 | 16 |
| | 50 | 32* |
| 5 | 10 | 27* |
| | 50 | 36* |
| 7 | 10 | ± 0 |
| | 50 | 12 |
| Solvent | - | ± 0 |

* Significantly differs from the control (solvent) ($P<0.05$).

**Table 5**. Anticoagulant activity and acute toxicity of the investigated compounds. (i.p. administration to white mice)

| Compound | Blood bleeding time | | Coagulation time | | $LD_{50}$ |
|---|---|---|---|---|---|
| | min | (%) | min | (%) | (mg/kg) |
| 1 | 1.65 ±0.20 | 137.5 | 2.88 ±0.30 | 128 | 447 |
| 2 | 1.33 ±0.15 | 110.8 | 2.85 ±0.33 | 127 | 515 |
| 3 | 1.75 ±0.28 | 145.8 | 3.00 ±0.35 | 133 | 325 |
| 4 | 1.80 ±0.18* | 150 | 3.07 ±0.30* | 136 | 410 |
| 5 | 1.30 ±0.14 | 108.3 | 3.40 ±0.34* | 151 | 355 |
| 6 | - | - | - | - | 355 |
| 7 | 1.36 ±0.14 | 113.3 | 3.10 ±0.32* | 137 | 708 |
| 8 | - | - | - | - | 515 |
| Control | 1.20 ±0.25 | 100 | 2.25 ±0.25* | 100 | |

* Significantly differs from the control ($P<0.05$).

**Table 6**. Effect of silyl(germyl) substituted isoxazolines and Allopurinol on ischaemia - reperfusion induced rhythm disturbances and lethality of rats.

| Compound | Dose (mg/kg, i.v.) | Rhythm Disturbances[1] | | Lethality[1] |
|---|---|---|---|---|
| | | Ventricular tachycardia | Ventricular fibrillations | |
| 1 | 5.0 | 9/10 | 7/10 | 3/10 |
| 2 | 2.0 | 9/10 | 6/10 | 1/10* |
| 3 | 2.0 | 10/10 | 8/10 | 3/10 |
| 4 | 2.0 | 11/12 | 7/12 | 1/12* |
| 5 | 2.0 | 9/10 | 4/10* | 0/10* |
| Allopurinol | 10.0 | 8/10 | 6/10 | 1/10* |
| Control (0.9% NaCl) | - | 15/15 | 14/15 | 7/15 |

[1] Ratio-incidence/number of animals in experiment;
* Significantly differs from the control (solvent) ($P<0.05$).

### *Pharmacology*

In experiments on noradrenaline-preconstricted blood vessel of a rabbit`s ear we investigated the vasodilating activity of the newly synthesized silyl and germyl isoxazolinyl substituted pyridines. Compounds ***2***, ***3***, ***4***, and ***5*** induced significant vasodilatation (***5*** being the most active). Compound ***7*** revealed weak vasodilation, but compound ***1*** induced 2-phase activity - vasodilatation/vasoconstriction (Table 4). Thus, we have found, that substitution of the silicon atom for germanium leads to the increase of vasodilating activity (***1***,***2***-***4***, ***5***), but the insertion of the methylene group between Ge and isoxazoline ring reduces vasodilating activity (compounds ***4*** and ***7***).

Table 5 summarizes the data on the anticoagulant activity of silyl(germyl)isoxazolines-2. In the experiments on mice the investigated compounds in general have no significant influence on the blood bleeding time (except compound ***4*** - prolongation of the bleeding time against control 150%).

It has been shown that all investigated germylisoxazolinyl substituted pyridines (***4***, ***5*** and ***7***) prolong the coagulation time (table 5), 3-(5`-triethylgermyl-3`-isoxazolinyl)pyridine hydrochloride (***5***) being the most active anticoagulant among all studied compounds. In this experiment silyl isoxazoline substituted pyridines (***1***-***3***) reveal weak anticoagulant activity.

In the experiments on the anasthetized rats ***2***, ***4***, ***5*** and Allopurinol protected animals from ischaemia - reperfusion induced lethality (table 6). Under our experimental conditions (15 min coronary artery occlusion with subsequent reperfusion) only the compound ***5*** protected heart from ventricular fibrillation.

In the experiments *in vitro* and *in vivo* it has been found that substitution of the silicon atom for the germanium one increases significantly the vasodilating and anticoagulant activity and leads to rise of cardioprotective properties of 3-pyridyl substituted isoxazolines. The insertion of the methylene group between Ge and the isoxazoline ring reduces the biological activity in the studied compounds.

It must be noted that germylisoxazolines ***4*** and ***7*** are also more potent (by 15-30 times) NO-inducers in HT-1080 (human lung fibrosarcoma) and MG-22A (mice hepatoma) cells than the silicon derivatives ***1*** and ***2***.

This group of compounds has a medium toxicity. The most toxic compound among all studied was 4-(5`-triethylsilyl-3`-isoxazolinyl)pyridine hydrobromide (325 mg/kg). The introduction of the methylene group between the triethylgermyl group and isoxazoline ring decreased the toxicity. 2-Pyridyl substituted isoxazolines are less toxic than 3- and 4-pyridyl substituted analogues. Triethylsilylisoxazolines are more toxic than germyl analogues.

### REFERENCES

1. E.Lukevics, L.Ignatovich, S.Germane, *Khim. Geterotsikl. Soed.*, 1995, ***10***, 1412.
2. E.Lukevics and L.Ignatovich, *Main Group Met. Chem.*, 1994, ***1***, 133.
3. F.Li, Z.Zhang, H.Gao, *Metal Based Drugs*, 1996, ***5***, 241.
4. J.K.Kolokolceva, V.N.Tshistokletov, A.A.Petrov, *Zn.Obsch.Khim.*,1969,***40***, 2612.
5. A.Padwa, J.G.MacDonald, *Tetrahedron Lett.*, 1982, ***23***, 3219.
6. A.Padwa, J.G.MacDonald, *J. Org. Chem.*, 1983, ***48***, 3189.
7. E.Lukevics, V.Dirnens, J.Popelis, A.Kemme, *J. Organomet. Chem.*, 1996, ***521***, 235.
8. E.Lukevics, V.Dirnens, P.Arsenyan, J.Popelis, A.Kemme, *Main Group Met. Chem.*, 1996, ***3***, 167.
9. E.Lukevics, V.Dirnens, N.Pokrovska, J.Popelis, A.Kemme, *Main Group Met. Chem.*, 1995, ***7***, 337.
10. E.Lukevics, V.Veveris, V.Dirnens, *Appl. Organomet. Chem.*, 1997, ***10-11***, 805.
11. R. Blattner, H.G. Classen, H. Dehnert and H.D. Doring, *Experiments on Isolated Smooth Muscle Preparations,* Hugo Sachs Elektronik KG, (BRD), 1980.
12. S.K.Wilson, O.S. Steinsland and S.H. Nelson, *J. Cardiovasc. Pharmacol.*, 1994, ***23***, 127.
13. Y. Thodorov, *Klinischeskie Laboratornye Issledovania v Pediatrii,* Medic. Phys., Sophia, 1966.

14. P. Castelli, A. M. Condemi, C. Brambillasca, P. Fundar , M. Botta, M. Lemma, P. Vanelli, C. Santoli, S. Gatti, E. Riva, *J. Cardiovasc. Pharmacol.,* 1995, ***25***, 119
15. R. Paul and S. Tchelitcheff, *Bull. Soc. Chim. Fr.*, 1962, 2215.
16. M. Kocevar, S. Polanc, M. Sollner, M. Tisler, and B. Vercek, *Synth. Commun.*, 1988, ***18***, 1427.
17. E.Lukevics, P.Arsenyan, S.Belyakov, J.Popelis, *J. Organomet. Chem.*, 1998, ***558***, 155.
18. E.Lukevics, P.Arsenyan, S.Belyakov, J.Popelis, *Main Group Met. Chem.*, 1998, ***21***, 557.
19. E.Lukevics, P.Arsenyan, *Khim. Heterotsikl. Soed.,* 1998, 564.

**Received: July 9, 1998 - Accepted: July 14, 1998**

